# Singlet oxygen imaging using fluorescent probe Singlet Oxygen Sensor Green in photosynthetic organisms

**DOI:** 10.1038/s41598-018-31638-5

**Published:** 2018-09-12

**Authors:** Ankush Prasad, Michaela Sedlářová, Pavel Pospíšil

**Affiliations:** 1 Department of Biophysics, Centre of the Region Haná for Biotechnological and Agricultural Research, Faculty of Science, Palacký University, Šlechtitelů 27, 783 71 Olomouc, Czech Republic; 2 Department of Botany, Faculty of Science, Palacký University, Šlechtitelů 27, 783 71 Olomouc, Czech Republic

## Abstract

Formation of singlet oxygen (^1^O_2_) was reported to accompany light stress in plants, contributing to cell signaling or oxidative damage. So far, Singlet Oxygen Sensor Green (SOSG) has been the only commercialized fluorescent probe for ^1^O_2_ imaging though it suffers from several limitations (unequal penetration and photosensitization) that need to be carefully considered to avoid misinterpretation of the analysed data. Herein, we present results of a comprehensive study focused on the appropriateness of SOSG for ^1^O_2_ imaging in three model photosynthetic organisms, unicellular cyanobacteria *Synechocystis* sp. PCC 6803, unicellular green alga *Chlamydomonas reinhardtii* and higher plant *Arabidopsis thaliana*. Penetration of SOSG differs in both unicellular organisms; while it is rather convenient for Chlamydomonas it is restricted by the presence of mucoid sheath of Synechocystis, which penetrability might be improved by mild heating. In Arabidopsis, SOSG penetration is limited due to tissue complexity which can be increased by pressure infiltration using a shut syringe. Photosensitization of SOSG and SOSG endoperoxide formed by its interaction with ^1^O_2_ might be prevented by illumination of samples by a red light. When measured under controlled conditions given above, SOSG might serve as specific probe for detection of intracellular ^1^O_2_ formation in photosynthetic organisms.

## Introduction

Singlet oxygen (^1^O_2_) formation is known as a distinctive functional response of organisms to stress stimuli. In plant cells, it has been linked mainly to high light^1–4^ but it has been known to occur also in other types of abiotic stress as high temperature, wounding or heavy metals^5^. At high light, ^1^O_2_ is formed by triplet-triplet energy transfer from triplet chlorophyll to molecular oxygen formed either by change in the spin orientation and charge recombination of the triplet radical pair^3^ [P680^•+^Pheo^•−^] in the antennae complex and the reaction centre of photosystem II (PSII)^6–8^. Under high temperature and wounding, ^1^O_2_ was shown to be formed by triplet-triplet energy transfer from triplet carbonyls to molecular oxygen formed during lipid peroxidation^9–11^. The role of ^1^O_2_ in plant organism is ambivalent; under moderate stress, ^1^O_2_ appears to act in retrograde signaling pathways^12–14^, whereas under severe stress, it causes oxidative damage of proteins and lipids^15–17^. At low ^1^O_2_ concentrations, retrograde signaling is associated with acclimation response, whereas at high ^1^O_2_ concentrations it is accompanied with programmed cell death^13^.

Several spectroscopic and microscopic techniques have been used to detect ^1^O_2_ formation in photosynthetic organisms both *in vitro* and *in vivo*^2,18,19^. Whereas *in vitro* ^1^O_2_ detection affords information on the quantification of ^1^O_2_ formation, *in vivo* techniques provide evidence on the spatiotemporal characterization of ^1^O_2_ within the plant cells. *In vitro* ^1^O_2_ detection comprises ^1^O_2_ phosphorescence, chemical trapping, and spin probe methods. Direct detection of ^1^O_2_ by phosphorescence at 1270 nm and chemical trapping based on O_2_ uptake due to the reaction of ^1^O_2_ with histidine or imidazole are feasible solely in isolated PSII reaction centres^3^. Spin probe technique based on the oxidation of diamagnetic nitroxide to paramagnetic nitroxyl radical detected by electron paramagnetic spectroscopy (EPR) is possible in PSII membranes, thylakoids and chloroplasts^20–22^. *In vivo* ^1^O_2_ detection includes cytochemical techniques using fluorescent and chemiluminescent probes combined with microscopic imaging^23^. In the last two decades, dansyl-based (DanePy)^24^ and Singlet Oxygen Sensor Green reagent® (SOSG)^9^ fluorescent probes based on the formation of endoperoxides were used to monitor ^1^O_2_ formation in photosynthetic organisms. These probes are composed of an anthracene moiety (electron donor) that quenches the fluorescence of the fluorochrome (electron acceptor) through electron transfer. Once the anthracene moiety traps ^1^O_2_, the resultant oxygen adduct fails to be functional intramolecular electron donor and the fluorescence is recovered.

Singlet Oxygen Sensor Green has been released and tested on plants since mid-2000’s^9,23^. Unlike other available fluorescent and chemiluminescent ^1^O_2_ probes, SOSG fulfils the requirement of high selectivity and specificity. It does not show an appreciable response to superoxide anion radical (O_2_^•−^) or hydroxyl radical (HO^•^) which is important especially in chloroplasts where a wide range of ROS are formed^4^. This fluorescein-based dye probe initially exhibits weak blue fluorescence at 395 and 416 nm, under excitation at 372 and 393 nm, respectively. Upon reaction with ^1^O_2_, the imminent product SOSG endoperoxide (SOSG-EP) provides green fluorescence, with the excitation and emission maxima lie, based on several authors, in the range of 504–508 nm and 525–536 nm, respectively^25–27^. Spectral properties of SOSG are similar to those of fluorescein which made this probe suitable for observation with a wide range of fluorescence and confocal microscope systems equipped with standard filters.

However, SOSG is known to have several drawbacks (unequal penetration and photosensitization) that must be taken into consideration. SOSG was introduced as a cell-impermeable probe but later studies shown its localization in most cell compartments under different stress conditions^9,23,28^. Further constraints of SOSG application in light-dependent studies is due to its photosensitization. It was demonstrated that SOSG can generate ^1^O_2_ under exposure to UV radiation (355 nm) and visible light (532 nm), respectively^25^. Later, it was shown that SOSG-EP can serve as photosensitizer even with much higher efficiency than SOSG^26^. A detailed study on SOSG photochemistry showed that singlet excited SOSG converts to triplet excited SOSG via intersystem crossing which further transfers the triplet excitation energy to molecular oxygen forming ^1^O_2_^27^.

Due to the SOSG and SOSG-EP photosensitization, an effort was initiated to discover fluorescent probe characterized by specificity and sensitivity similar to SOSG; however, lacking its negative trait of photosensitization^25^. Pedersen and co-workers discovered and applied on mammalian cells, a modified ^1^O_2_ indicator called Aarhus Sensor Green (ASG) (a tetrafluoro-substituted fluorescein derivative covalently linked to a 9,10-diphenyl anthracene moiety)^29^. Aarhus Sensor Green is characterized by spectral properties similar to SOSG omitting its negative trait of photosensitization. Nevertheless, ASG has neither been tested on plants nor available for routine applications. Thus developing new ^1^O_2_ sensors remained a goal of research teams. In chemical systems, more alternatives to SOSG exist, including FRET-based ratiometric monitoring of ^1^O_2_ with acene-doped conjugated polymer nanoparticles^30^ or ^1^O_2_ detection in solutions by Bodipy-based energy transfer cassette^31^. Recently, Tang *et al*.^32^ proposed an alternative indocyanine green (ICG), a near-infrared (NIR) tricarbocyanine probe that is decomposed after reaction with ^1^O_2_. However, the absorption and emission peaks of ICG at ~807 nm and ~822 nm, respectively, make it less convenient for the fluorescence microscopy.

The present study deals with the re-examination of SOSG properties for its possible utilization in model photosynthetic organisms (cyanobacteria, algae, and higher plants) under high light. Penetration of SOSG into the cells can be enhanced by mild heating (cyanobacteria) or moderate-pressure tissue infiltration (higher plants) under conditions avoiding ^1^O_2_ formation by high temperature or leaf wounding itself. Illumination by a red light (λ ≥ 600 nm) is advised to avoid ^1^O_2_ formation due to SOSG and SOSG-EP photosensitization. As the experimental conditions may significantly affect the obtained results, we attempt to summarize the principal factors that must be taken into account when imaging ^1^O_2_ with SOSG in photosynthetic organisms.

## Material and Methods

### Plant material

*Synechocystis* sp. (PCC 6803) was grown in BG-11 medium supplemented with 5 mM glucose and 5 mM sodium bicarbonate as the main carbon source under a continuous white light (100 μmol photons m^−2^ s^−1^) at 25 °C^33^. The cell culture was permanently stirred using magnetic stirrer RT 5 power (IKA Werke GmbH, Staufen, Germany) to obtain a constant CO_2_ concentration in the growth medium. The cells from the end of log phase at a density of approximately 10^8^–10^9^ cells ml^−1^ were used for experiments. *Chlamydomonas reinhardtii* (CC-002) was obtained from the Chlamydomonas Genetic Center (Duke University, Durham, NC, USA). The cells were cultivated in Tris-Acetate-Phosphate (TAP) medium or in Bold’s basal medium (BBM) in a continuous white light (100 μmol photons m^−2^ s^−1^) in Algaetron AG 230 (Photon Systems Instruments, Drásov, Czech Republic). The growth was achieved under permanently stirred condition using shaker (Orbital Shaker PSU-10i, Biosan, Riga, Latvia) to obtain a constant CO_2_ concentration in the growing medium^10,34^. The cells were studied at a density of approximately 7 × 10^7^ cells ml^−1^ during the stationary growth phase. *Arabidopsis thaliana* (Col-0) seeds from the Nottingham Arabidopsis Stock Centre (NASC) were soaked in distilled water for 4 days at 4 °C and transferred into the growing pots filled with a peat substrate (Klasmann, Potground H). The plants were grown for 4–6 weeks in a growing chamber at a photoperiod of 8/16 h light/dark (100 μmol photons m^−2^ s^−1^) at a temperature of 22 °C/20 °C, respectively and relative humidity 60%.

### SOSG staining

Synechocystis cells (BG-11 medium, pH 7.1), Chlamydomonas cells (TAP medium, pH 7.0 or BBM medium pH 6.8) and Arabidopsis leaf pieces with adaxial side of leaf up (5 × 5 mm, HEPES pH 7.5) were placed in 2 ml Eppendorf tube and treated with 50 μM SOSG (Molecular Probes Inc., Eugene, OR, USA) for 30 min either in dark or exposed to light. For Synechocystis and Chlamydomonas cells, the media containing SOSG was replaced with growing medium before measurement while for Arabidopsis leaf, the measurement was done in pure buffer.

### High light

The illumination was performed using a LED source with a light guide CL6000 LED Zeiss (Carl Zeiss Microscopy GmbH, Jena, Germany). Samples were exposed to continuous white light (λ = 400–750 nm) or red light (λ ≥ 600 nm) with an intensity of 1000 μmol photons m^−2^ s^−1^ transferred by optical fiber guide to the top of Eppendorf tube. Long-pass edge interference filter (600 nm) (Andover Corporation, Salem, NH, USA) was used to cut off the blue-green region of the spectra.

### Fluorescence emission spectra

Fluorescence emission spectra were measured using a fluorescence spectrometer F-4500 (Hitachi, Tokyo, Japan). For fluorescence emission spectra, the SOSG was excited at 488 nm and SOSG-EP fluorescence was recorded in the spectral range of 500–640 nm.

### Singlet oxygen imaging by confocal laser scanning microscopy

Immediately after staining, the cells were transferred to corresponding fresh media and visualized by confocal laser scanning microscopy (Fluorview 1000 unit attached to IX80 microscope; Olympus Czech Group, Prague, Czech Republic). The excitation of SOSG was performed by a 488 nm line of an argon laser and the emission was detected by a 505–525 nm filter. Cell morphology was visualized by transmitted light detection module with 405 nm excitation using a near ultraviolet (405 nm) diode laser and differential interference contrast (DIC) filters. The proper intensity of all lasers was set according to unstained samples at the beginning of each experiment^35^. Integral distribution of signal intensity (0–4096) in 12-bit microphotographs was evaluated by image analysis software FV10-ASW 4.0 Viewer (Olympus).

## Results

### SOSG penetration

**Figure 1:**
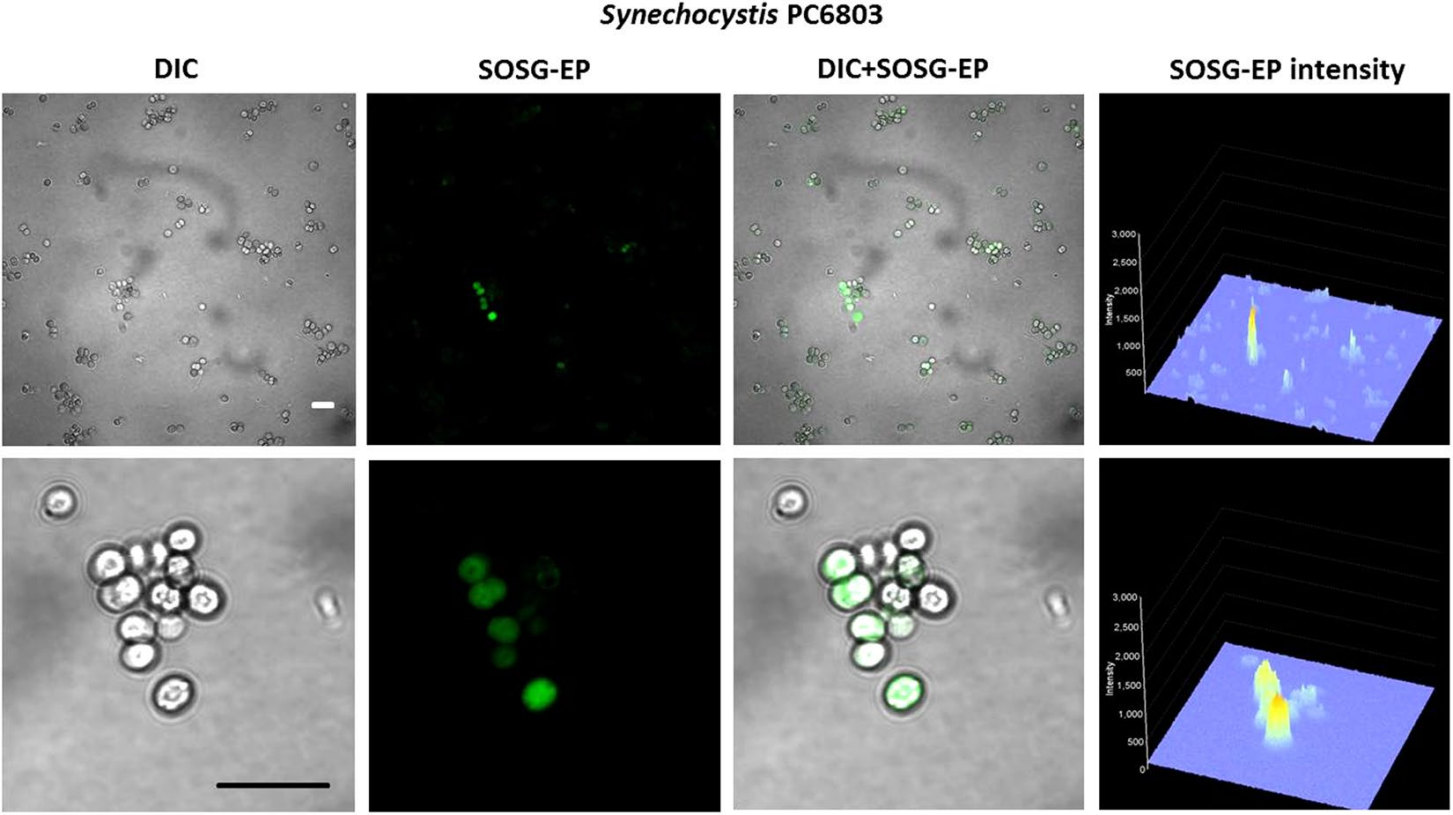
Singlet oxygen imaging in Synechocystis cells. Synechocystis cells were exposed to red light for 30 min in the presence of 50 μM SOSG and at 37 °C. From left to right: Nomarski DIC, SOSG-EP fluorescence (λem = 505–525 nm) and combined channel (DIC + SOSG-EP). Bar in both the upper and the lower panel represents 5 µm.

**Figure 2:**
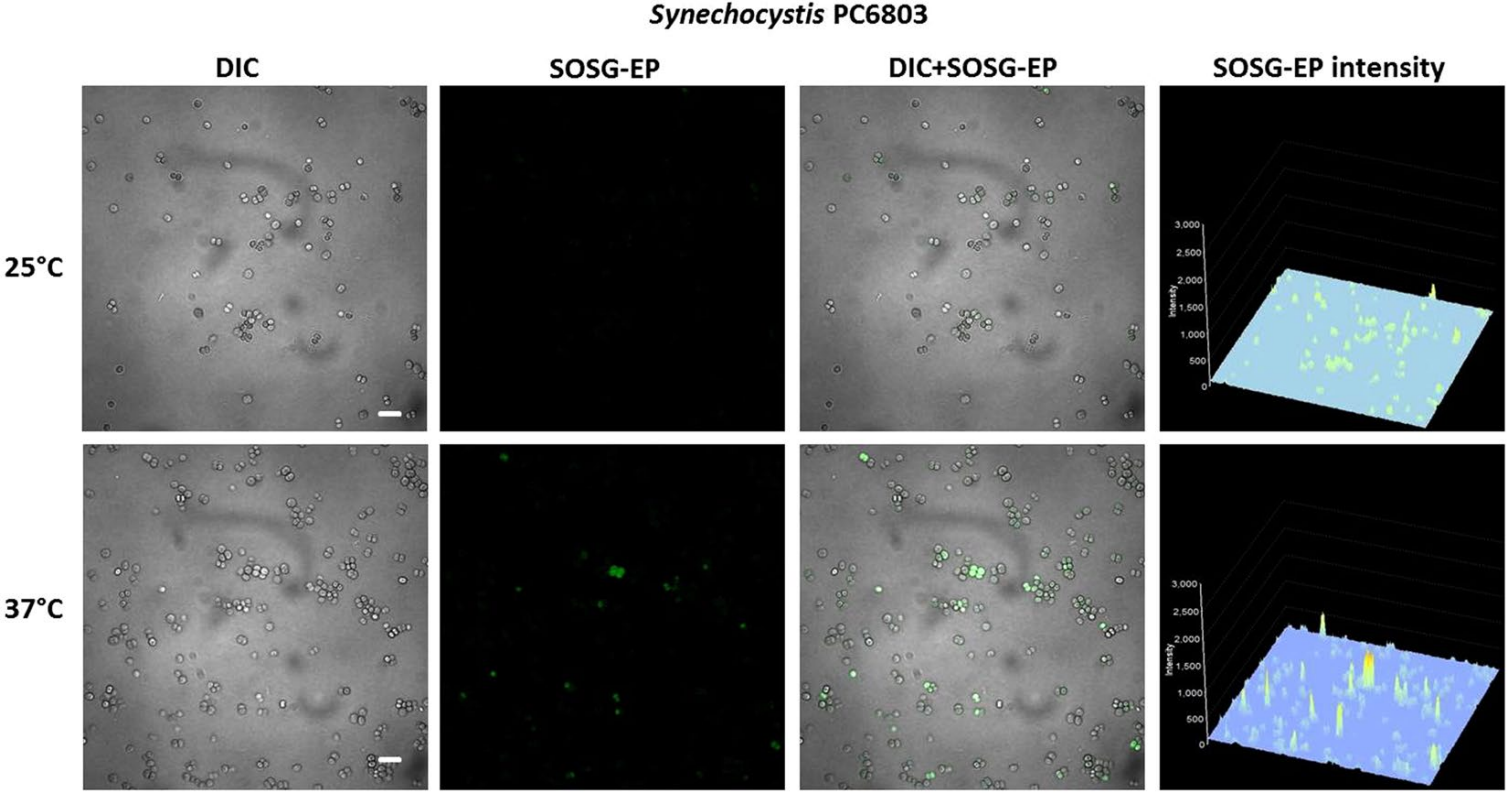
Effect of mild heating on the penetration of SOSG into the Synechocystis cells. Synechocystis cells were exposed to red light for 30 min in the presence of 50 μM SOSG at the temperature (25 °C) (upper panel) and (37 °C) (lower panel). From left to right are Nomarski DIC, SOSG-EP fluorescence (λem = 505–525 nm) and combined channel (DIC + SOSG-EP). Bar represents 5 µm.

**Figure 3:**
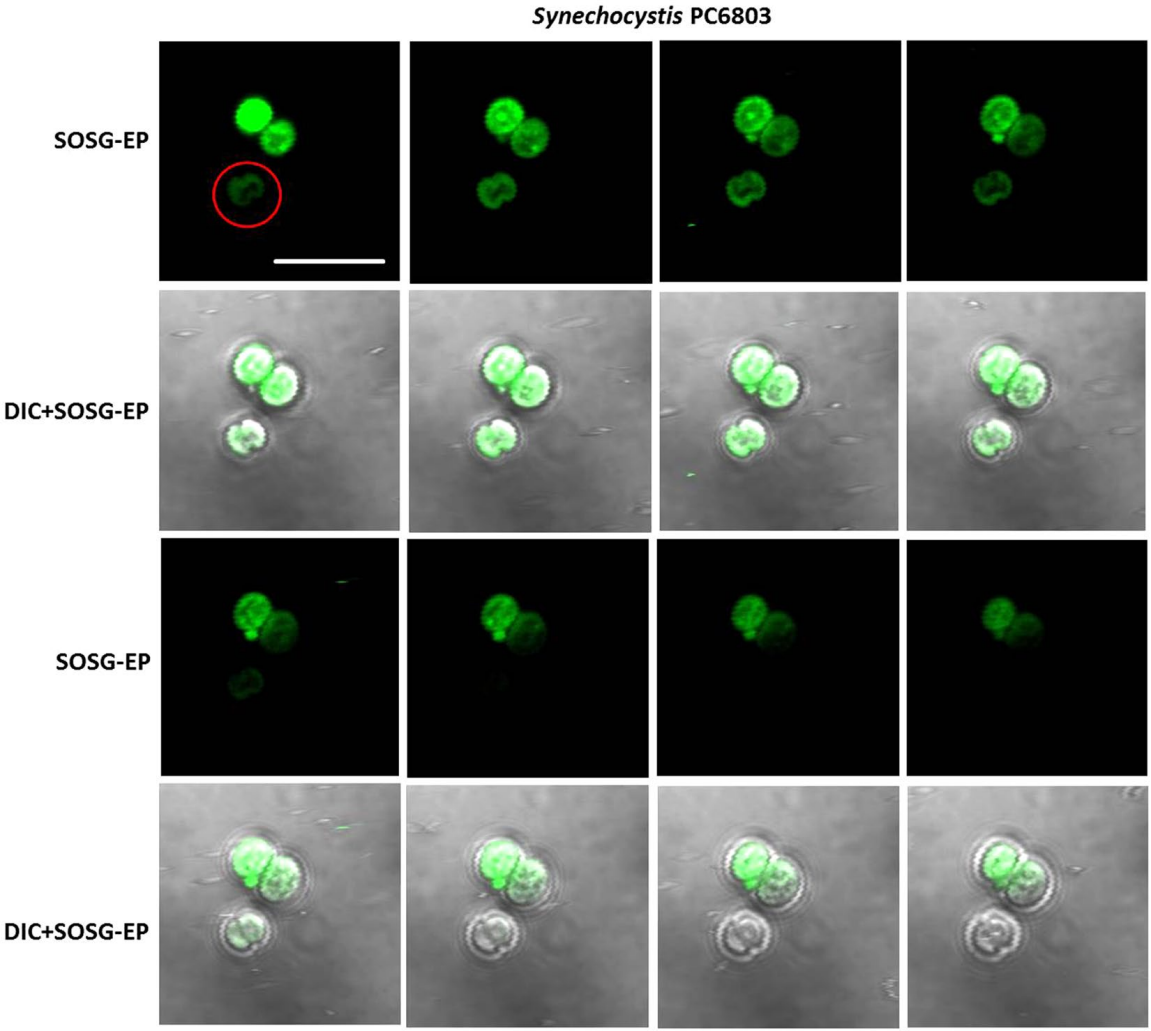
Series of optical sections through Synechocystis cells. Red circle shows SOSG-EP fluorescence at the outer regions of the cell. SOSG-EP and DIC + SOSG-EP channel channels are shown. Bar represents 5 µm. Other experimental conditions as in Fig. 1.

**Figure 4:**
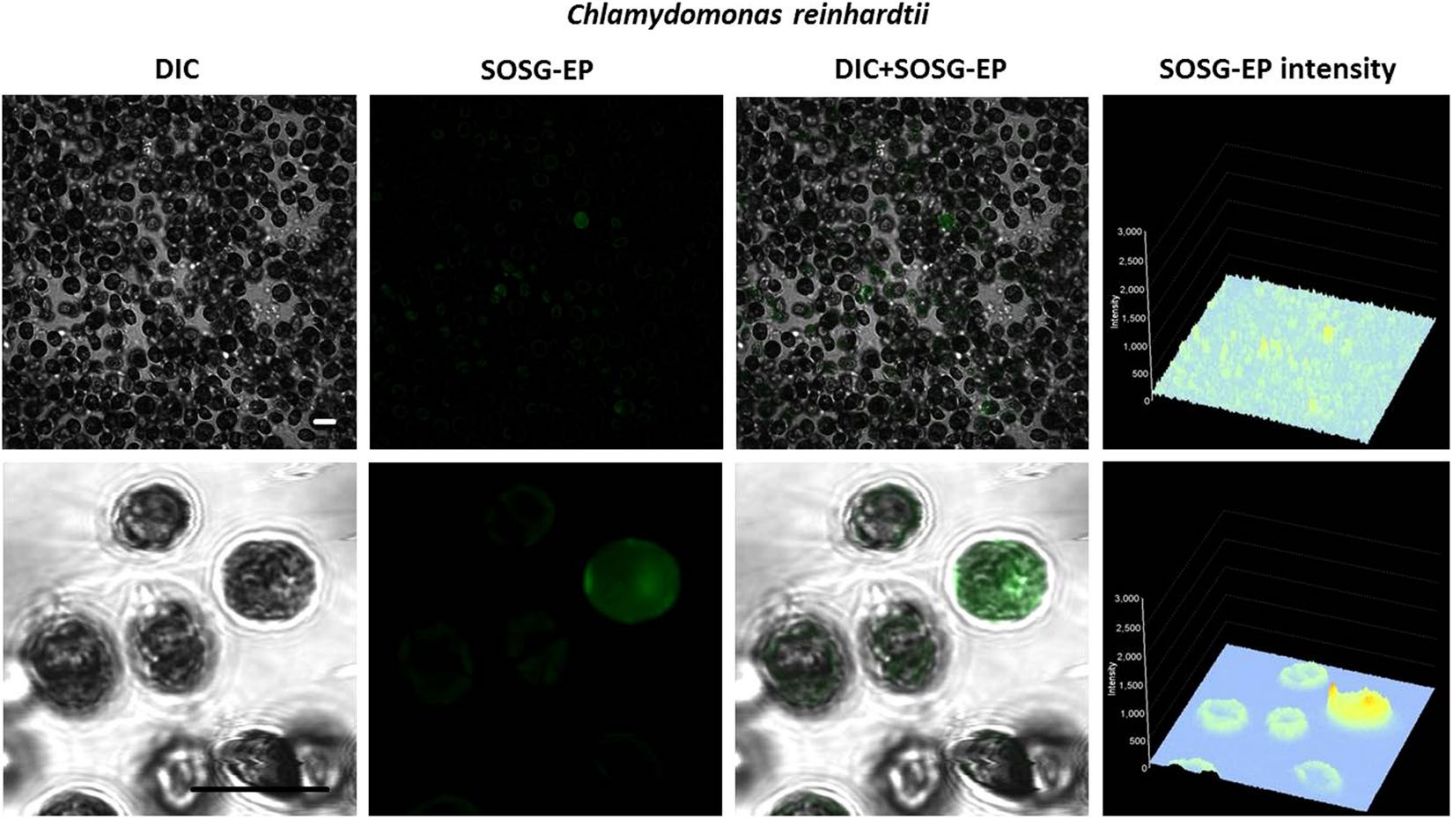
Singlet oxygen imaging in Chlamydomonas cells. Chlamydomonas cells were exposed to red light for 30 min in the presence of 50 μM SOSG. From left to right: Nomarski DIC, SOSG-EP fluorescence (λem = 505–525 nm) and combined channel (DIC + SOSG-EP). Bar represents 10 µm.

To monitor ^1^O_2_ formation in unicellular photosynthetic organisms, SOSG-EP fluorescence was detected in Synechocystis (Figs 1–3) and Chlamydomonas (Fig. 4) cells by confocal laser scanning microscopy. Figures 1 and 4 demonstrate Nomarski DIC channel (greyscale), fluorescence channel (green), the combination of both channels and the integral distribution of the signal intensity within the sample. When Synechocystis cells were exposed to red light in the presence of SOSG, SOSG-EP fluorescence was observed. Detail focus shows that SOSG-EP fluorescence is distributed uniformly in the whole cell (Fig. 1). Similarly, exposure of Chlamydomonas cells to red light resulted in the appearance of SOSG-EP fluorescence localized predominantly within the chloroplasts (Fig. 4). Whereas the penetration of SOSG into the Chlamydomonas cells has no serious limitation and uptake can be achieved by the placement of cells in the dye, the penetration of SOSG into the Synechocystis cells is restricted due to the presence of polysaccharide matrix forming mucous surface structures. Figure 2 shows that SOSG penetration into the Synechocystis cells is improved largely by mild heating (37 °C) known to dissolve mucoid sheath. As mild heating of Synechocystis cells did not enhanced SOSG-EP fluorescence in dark (data not shown), it indicates that mild heating to 37 °C causes no ^1^O_2_ formation by lipid peroxidation initiated by lipoxygenase as previously shown in Chlamydomonas exposed to 40 °C^10^. Figure 3 (red circle) shows that some cells exhibit SOSG-EP fluorescence from the outer regions of the cell. A series of optical sections through the cells reveals that SOSG is uniformly spread out in these cells and SOSG-EP fluorescence from the outer regions of the cell is due to the plane of focus. In order to confirm that observed SOSG-EP fluorescence enhancement is due to ^1^O_2_ generation, the effect of scavengers of ^1^O_2_ (sodium azide/histidine) was measured in Chlamydomonas cells exposed to red light. A considerable suppression in SOSG-EP fluorescence was observed (see Supplementary Figs S4–S5).

**Figure 5:**
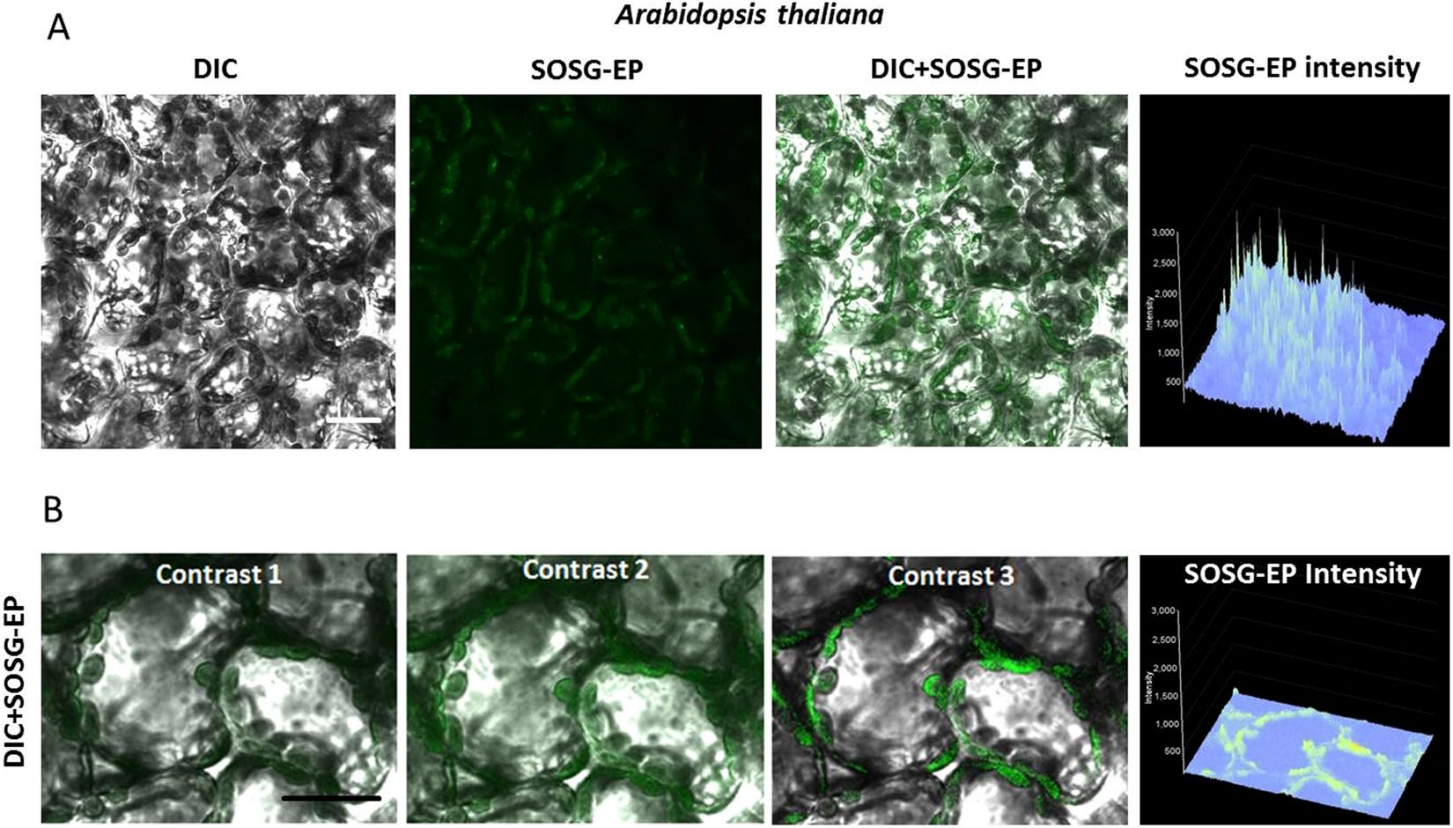
Singlet oxygen imaging in Arabidopsis leaves. (**A**) Arabidopsis leaves were exposed to red light for 30 min in the presence of 50 μM SOSG. From left to right: Nomarski DIC, SOSG-EP fluorescence (λem = 505–525 nm) and combined channel (DIC + SOSG-EP). (**B**) Example of post-processing signal-to-noise ratio applied to a 12-bit figure (Arabidopsis, 50 uM SOSG, 30 min, red light, DIC + SOSG-EP fluorescence channel at 505–525 nm). SOSG-EP fluorescence was emphasized by limiting shades to 0–1500 or 50–1000 and increasing contrast of SOSG-EP channel from 1, 2 and 3. Bar represents 20 µm.

**Figure 6:**
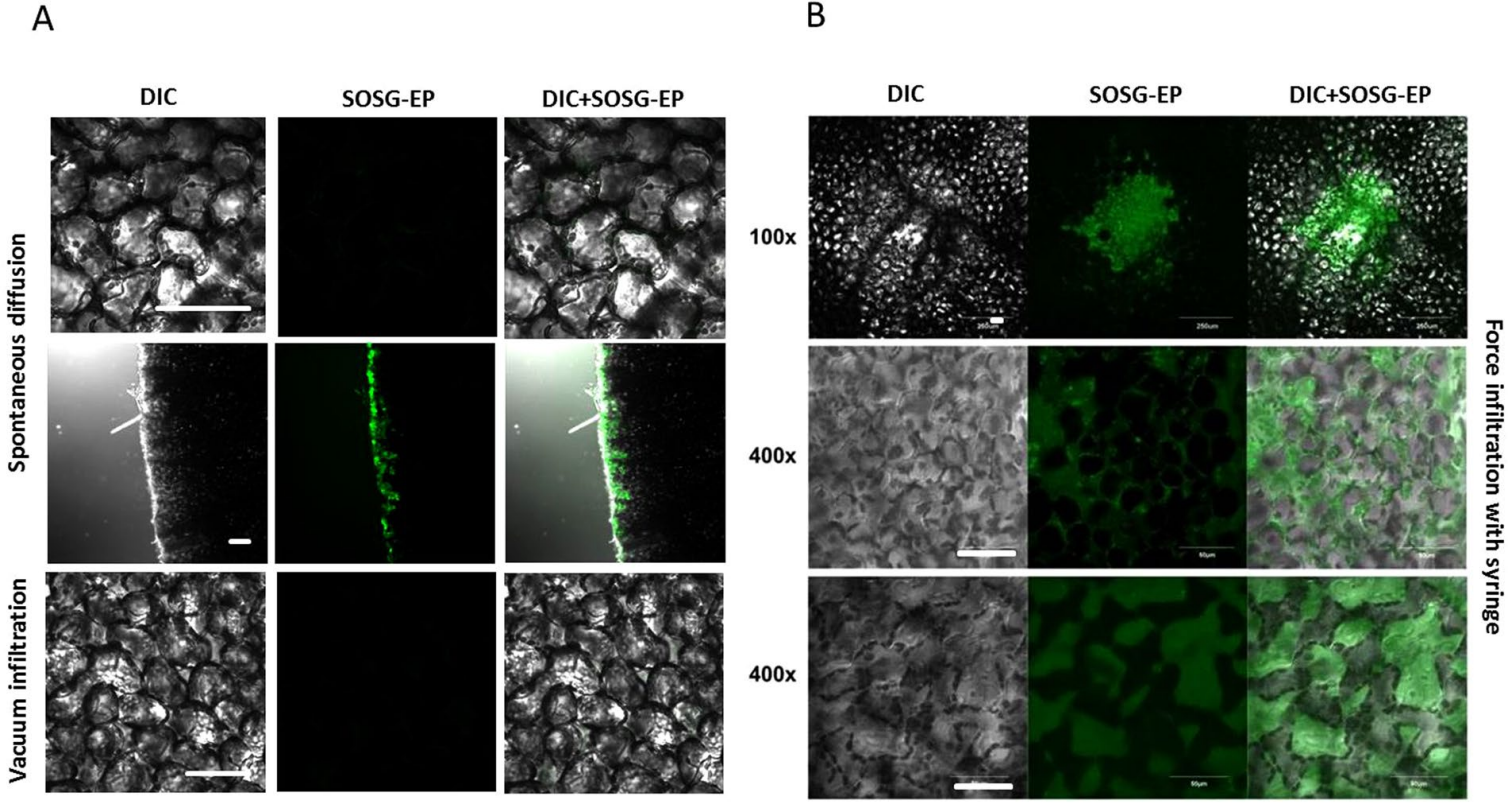
SOSG infiltration into Arabidopsis leaves. (**A**) Spontaneous diffusion measured at the centre of the excised leaf (upper row), spontaneous diffusion measured at the cut edges (middle row) and vacuum infiltration (lower row). (**B**) Force infiltration with a syringe. The image with resolution 100x shows the mechanical injury at the site of force infiltration and 400x magnification are focused on layers of sponge and below on palisade parenchyma cells. In A and B, from left to right are Nomarski DIC, fluorescence (λem = 505–525 nm) and combined (DIC + SOSG) channel. Bar represents 50 µm.

Light-induced formation of ^1^O_2_ was accordingly visualized with SOSG in Arabidopsis leaves (Fig. 5). When Arabidopsis leaves were exposed to red light in the presence of SOSG, the SOSG-EP fluorescence was detected predominantly from the chloroplasts located at the periphery of the mesophyll cells (Fig. 5A). Figure 5B shows a series of SOSG-EP fluorescence at limiting shades [0–1500 or 50–1000] and increasing contrast of SOSG-EP channel [contrast range: 1–3]. In Arabidopsis leaves, SOSG penetration is limited due to the tissue complexity comprising the upper and lower epidermis covered by cuticle. Penetration of SOSG into the mesophyll cells can be achieved through cutting edge (to a less extent by stomata) by pressure infiltration using the shut syringe. The efficiency of SOSG penetration into the mesophyll cells depends on several factors including the leaf age and water content within the tissue (data not shown). This approach is efficient in the uniform delivery of SOSG into the mesophyll cells and gentle with respect to mechanical injury known to cause ^1^O_2_ formation as previously shown in Arabidopsis leaves^11^. Different approaches such as spontaneous diffusion measured either at the centre of the excised leaf or at cut edges and vacuum infiltration were used; however, no penetration or uneven penetration was possible to be achieved (Fig. 6A). Furthermore, force infiltration with syringe results in the uneven delivery of SOSG to the mesophyll cells and the leaf injury at the site of puncture. Figure 6B shows that penetration into the tissues is inconsistent, randomly distributed in the both intercellular and extracellular spaces. The mechanical injury provides the highest signal in the centre (100x magnification) and influences results of staining upon stress treatment (at 400x magnification focused on layers of sponge and below on palisade parenchyma cells).

**Figure 7:**
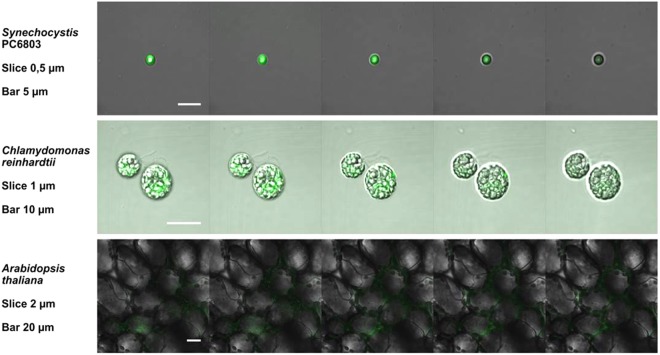
Series of optical sections through cells of Synechocystis, Chlamydomonas, and tissues of Arabidopsis leaves. Other experimental conditions as in Figs 1, 4 and 5.

To confirm that SOSG penetrates uniformly into the cells, 3-D distribution of SOSG-EP fluorescence within cells or tissues was visualized by Z-stacks of thin optical sections. Figure 7 shows a series of 5 optical sections with a distance of 0.5 µm (Synechocystis cells), 1 µm (Chlamydomonas cells) and 4 µm (Arabidopsis leaves), respectively. SOSG-EP fluorescence measured in the multiple layers in Synechocystis and Chlamydomonas cells were observed from overall cell volume with fluorescence intensity higher at the cell periphery. In Arabidopsis leaves, SOSG-EP fluorescence comes predominantly from the chloroplasts located at the periphery of the cells. These results show that penetration of the probe (at the depth of cells and tissues) can be reached with a proper modified procedure of staining.

### SOSG photosensitization

**Figure 8:**
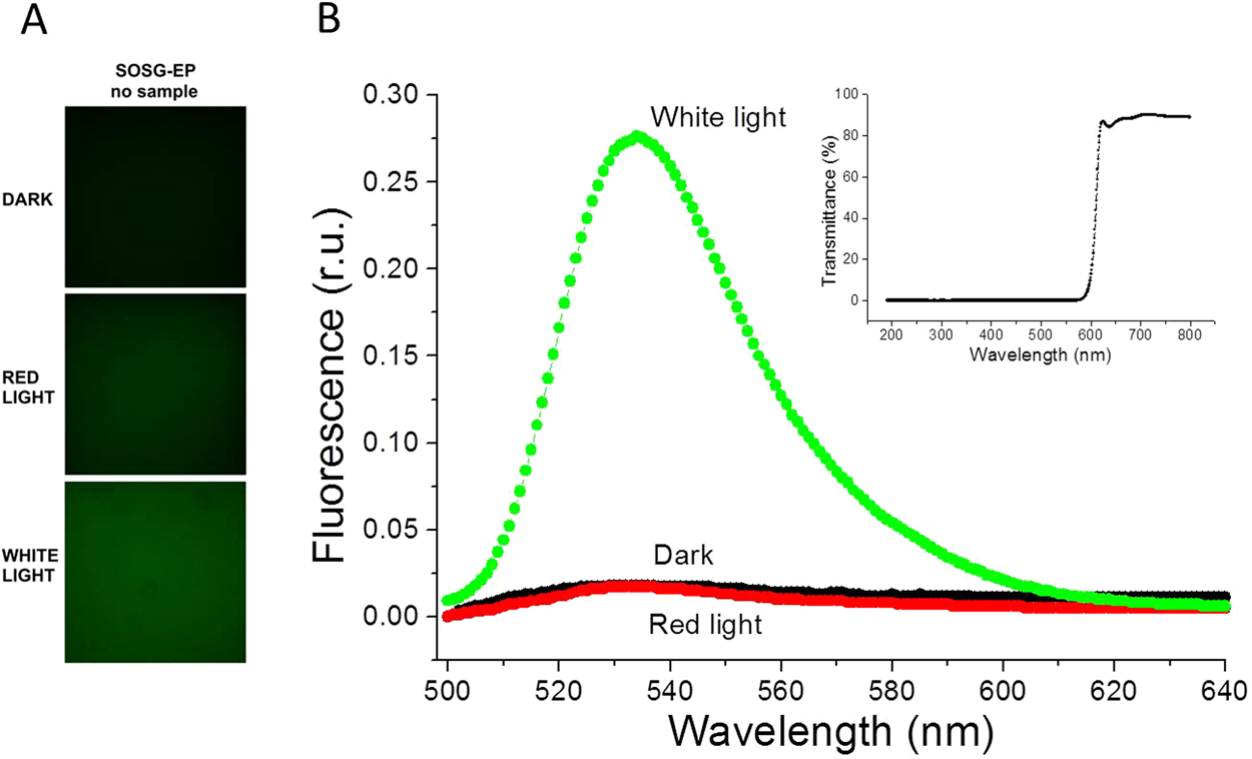
Effect of red and white light on SOSG photosensitization. (**A)** SOSG/SOSG-EP fluorescence after exposure of SOSG to red/white light monitored by confocal laser scanning microscopy. **(B**) SOSG/SOSG-EP fluorescence emission spectra after exposure of SOSG to red or white light measured using fluorescence spectrometer. In A and B, SOSG (50 µM in A and 1 µM in B) was kept in dark or illuminated either with red (≥600 nm) or white light (400–750 nm) (1000 µmol photons m^−2^ s^−1^) for 30 min in the absence of any biological sample. SOSG/SOSG-EP fluorescence was produced by excitation at 488 nm and emission was measured in spectral range 505–525 nm (**B**) and 500–640 nm (**B**). SOSG was dissolved in 40 mM HEPES buffer (pH 7.5) containing 1% methanol. Insert shows transmission spectrum of long-pass edge filter measured using Specord 250 Plus (Analytik Jena AG, Germany).

**Figure 9:**
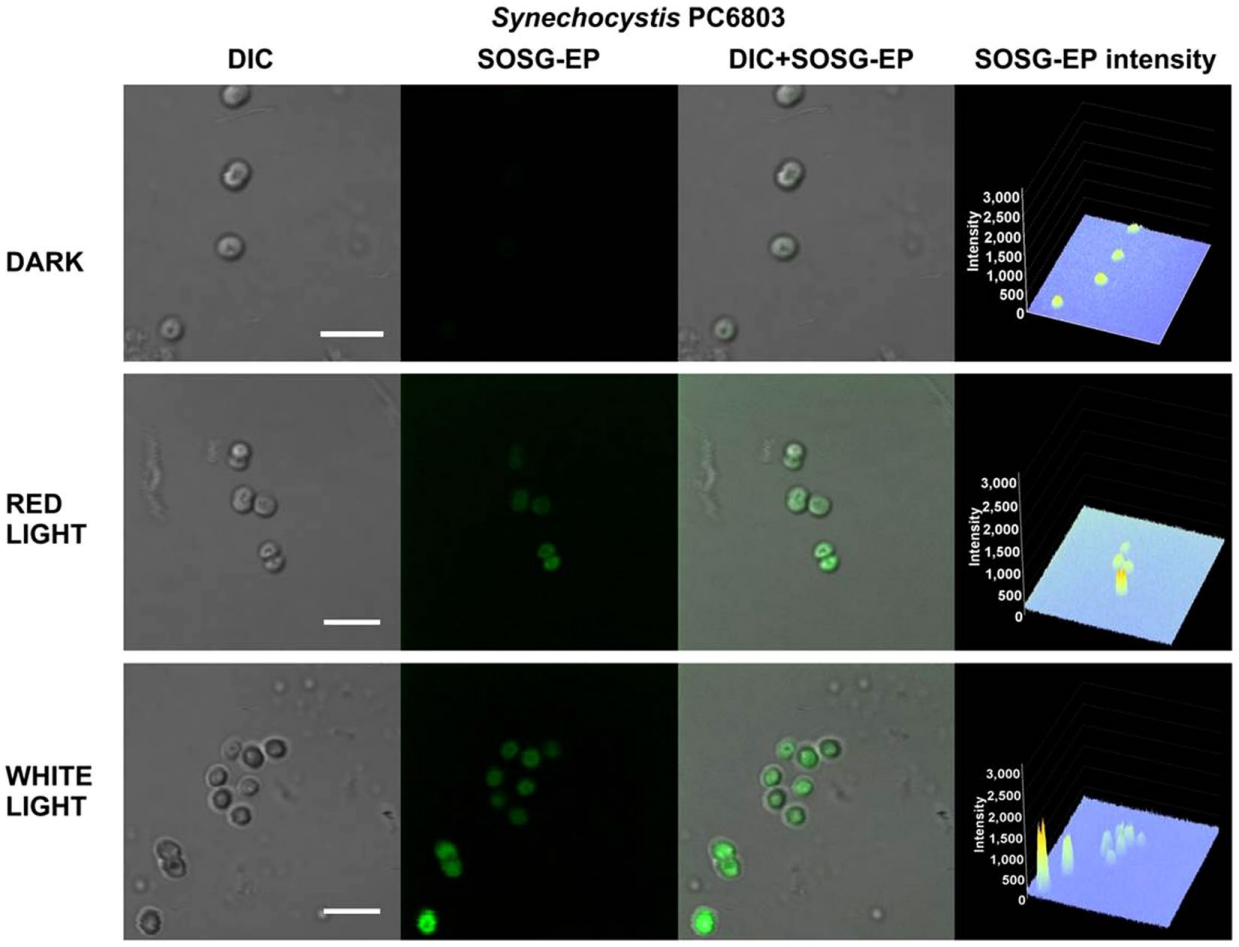
Effect of red and white light on singlet oxygen imaging in Synechocystis cells. Synechocystis cells were treated in 50 μM SOSG for 30 min at 37 °C either in dark or exposed to red/white light. For each treatment following images are presented (from left to right): Nomarski DIC, SOSG-EP fluorescence (λem = 505–525 nm), combined channel and integral distribution of the signal intensity within the sample (Z-axis represents the levels of brightness for each pixel, ranging between 0 and 3200). Bar represents 5 µm.

**Figure 10:**
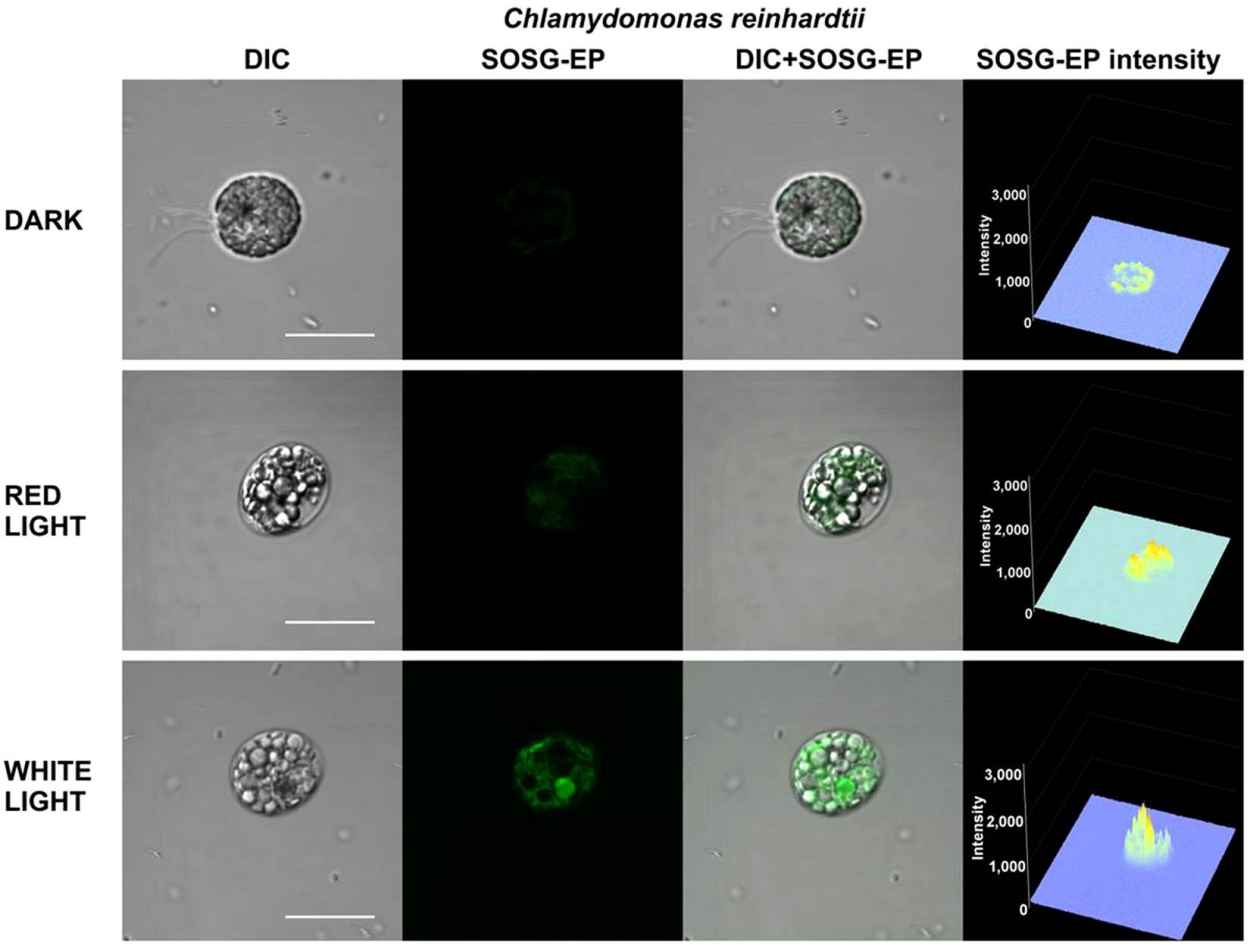
Effect of red and white light on singlet oxygen imaging in Chlamydomonas cells. Chlamydomonas cells were treated in 50 μM SOSG for 30 min either in dark or exposed to red/white light. For each treatment following images are presented (from left to right): Nomarski DIC, SOSG-EP fluorescence (λem = 505–525 nm), combined channel and integral distribution of the signal intensity within the sample (Z-axis represents the levels of brightness for each pixel, ranging between 0 and 3200). Bar represents 10 µm.

**Figure 11:**
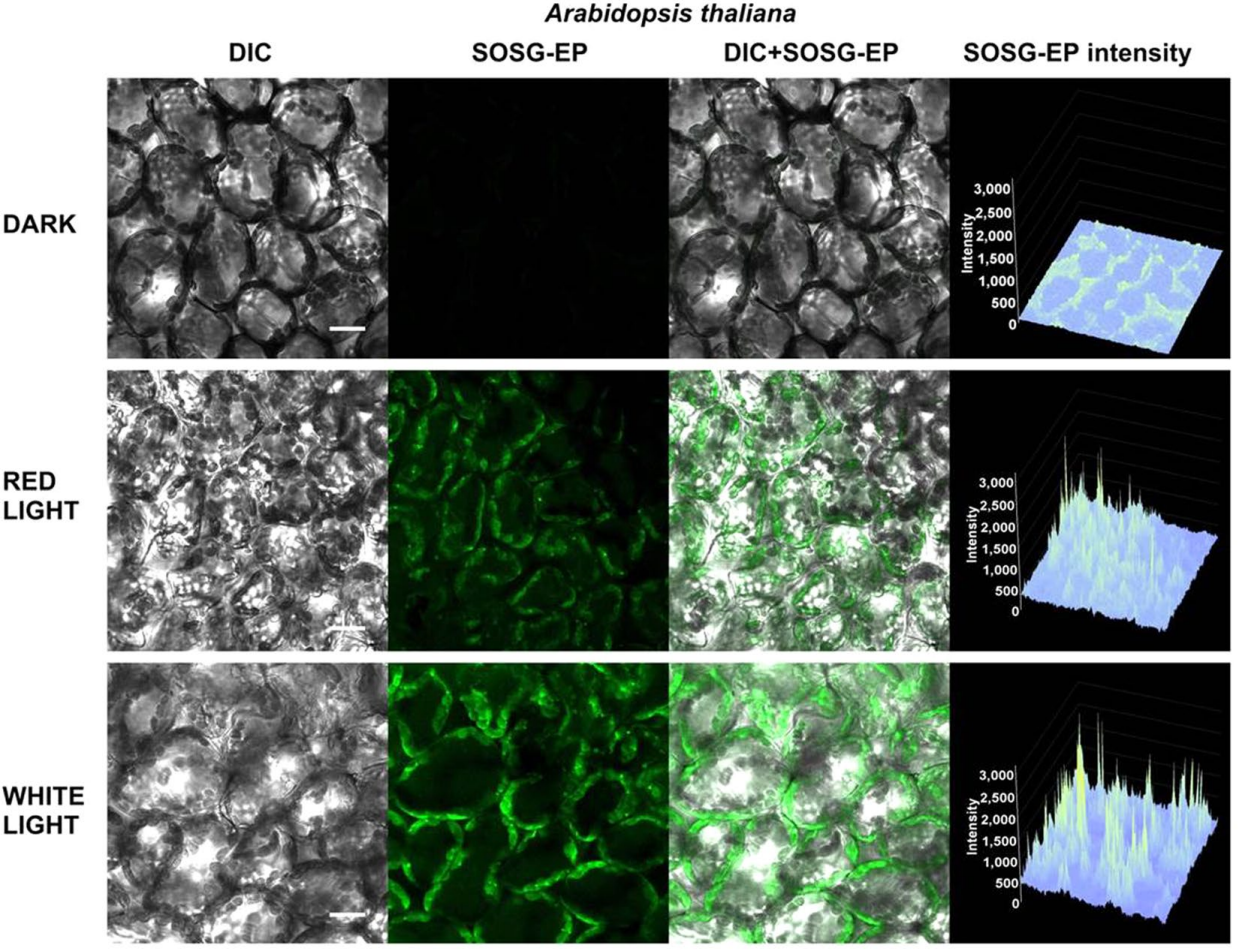
Effect of red and white light on singlet oxygen imaging in Arabidopsis leaves. Arabidopsis leaves were treated in 50 μM SOSG for 30 min either in dark or exposed to red/white light. For each treatment following images are presented (from left to right): Nomarski DIC, SOSG-EP fluorescence (λ_em_ = 505–525 nm), combined channel and integral distribution of the signal intensity within the sample (Z-axis represents the levels of brightness for each pixel, ranging between 0 and 3200). Bar represents 20 µm.

To test the photosensitization of SOSG and SOSG-EP, the probe was first illuminated in the absence of sample either with red (λ ≥ 600 nm) or white (400–750 nm) light (Fig. 8). Figure 8A shows SOSG/SOSG-EP fluorescence detected by confocal laser scanning microscopy. When SOSG was kept in dark or exposed to red light, negligible SOSG-EP fluorescence was detected. On the contrary, exposure of SOSG to white light resulted in pronounced SOSG-EP fluorescence. Figure 8B shows SOSG/SOSG-EP fluorescence spectra with an emission maximum at 535 nm measured using fluorescence spectrometer. When SOSG was exposed to white light, SOSG-EP fluorescence was several folds higher compared to red light. Based on these results, it is concluded that red light did not cause ^1^O_2_ formation due to the photosensitization of SOSG and SOSG-EP, whereas white light resulted in SOSG and SOSG-EP photosensitization. In further, SOSG was illuminated either with red or white light in Synechocystis cells (Fig. 9), Chlamydomonas cells (Fig. 10) and Arabidopsis leaves (Fig. 11) (also see Supplementary Figs S1–S3). In the dark, negligible SOSG-EP fluorescence was due to excitation of SOSG probe by a laser beam, ^1^O_2_ formation by excitation of photosynthetic pigments by a laser beam and/or dark ^1^O_2_ formation due to the spontaneous oxidative processes. When Synechocystis (Fig. 9) and Chlamydomonas (Fig. 10) cells were exposed to white light, SOSG-EP fluorescence was enhanced as compared to red light. Similarly, when Arabidopsis leaves were exposed to white light, the intensity of SOSG-EP fluorescence was higher compared to red light (Fig. 11). These results show that exposure of SOSG to white light causes SOSG and SOSG-EP photosensitization.

## Discussion

In the current study, the use of SOSG for monitoring of ^1^O_2_ formation was tested in three model photosynthetic organisms. It can be mentioned that despite being tested for numerous applications in the last decade, the exact composition of this probe was not known during initial 5 years of its utilization. Later, Ragas with co-workers showed a combination of fluorescein and anthracene moieties in SOSG by liquid chromatography and UV-VIS spectra, complemented with mass spectrometry and NMR spectroscopy^25^. As the use of SOSG for detection of ^1^O_2_ formation has a restriction regarding the probe penetration into the cells and probe photosensitization, it is crucial to control the uniform distribution of SOSG in the cells and prevent SOSG photosensitization^36^.

### SOSG permeability

Penetration of fluorochromes into the plant cells has been a matter of discussions^23,37,38^. SOSG has been claimed as a cell-impermeant substance by the producer^39^; however, experiences acquired during the last decade of its application on biological samples by several research groups proved that it is uptaken by animal cells^26,40^. Unlike animal cells, the presence of cell wall in combination with additional mechanical barriers containing polysaccharide sheath enclosing in the cyanobacteria or cuticle waxes in the plant might hinder delivery of the fluorochrome to the precise organelles of plant cells^38^. Since no diacetate form of SOSG has been available to ease the probe uptake through the plasma membrane, a relatively high concentration (50–250 µM) and longer incubation periods (30 min) has to be used. Since Flors and co-workers^9^ reported the applications of SOSG on diatom species and Arabidopsis leaves, several teams employed SOSG in their experiments with unicellular organisms^41^ and higher plant^42–46^. SOSG-EP fluorescence visualized in the multiple optical sections through cells of Synechocystis, Chlamydomonas and Arabidopsis confirmed that SOSG penetrates into cells in all tested model photosynthetic organisms (Figs 3 and 7). However, it should be pointed here that confocal laser scanning microscopy has to be used to prove the proper even penetration of the probe into the cells. Detection of ^1^O_2_ formation exclusively by fluorescence spectrometer might lead to the detection of SOSG-EP fluorescence originating from the extracellular space and thus can be lead to a false signal and misinterpretation of the analysed data.

#### SOSG permeability in unicellular organisms

It is of note that some authors reported that SOSG does not penetrate inside the intact Synechocystis cells^47,48^. Synechocystis cells are known to excrete polysaccharides to form an outermost slimy layer during cell growth^49^. Penetration of SOSG into the cells enclosed by capsule and mucus might represent a mechanical barrier for SOSG penetration into the Synechocystis cells. In this study, we clearly demonstrated that using active cells from the log phase of the growing curve combined with an increase in temperature (37 °C for ca 15 min) as used previously^47^ can substantially increase the uptake of SOSG (Figs 1 and 2). It is proposed here that secreting the mucus out of Synechocystis cells can decrease SOSG infiltration at later stages of cultivation in liquid media or on agar.

#### SOSG permeability in higher plants

Conversely, higher plants with multiple types of cells and tissues represent a significantly greater challenge. Based on our experiences, the effort to detect intracellular generation of ^1^O_2_ in multicellular plants can be complicated by uneven staining or mechanical injury during handling with samples. It should be also noted that O_2_, CO_2_ and other gases produced by the metabolism of living tissue create microbubbles within intercellular space which counteract with uniform sensor delivery to all cells. We have tested several approaches for SOSG infiltration to tissues of Arabidopsis leaves comprising spontaneous diffusion, vacuum infiltration, force infiltration with a syringe and pressure infiltration using the shut syringe. Spontaneous diffusion in centre of the excised leaf based on transpiration stream (Fig. 6A, upper row) and vacuum filtration (Fig. 6A, lower row) were found to be with no or uneven distribution of the fluorescent probe. Spontaneous diffusion at cut edges allows uniform distribution of SOSG within several layers of cells from the cut edge (Fig. 6A, middle row). However, the light-induced formation of ^1^O_2_ was largely overlapped by ^1^O_2_ formed due to the mechanical injury at the cut edge. It has been recently demonstrated that ^1^O_2_ is formed by lipid peroxidation initiated by lipoxygenase at the cut edge in the Arabidopsis wounding response^11^. The use of spontaneous diffusion at cut edge is possible when 1–3 layers of cells on the cut edge with ^1^O_2_ formation due to the mechanical injury is excluded from the evaluation. Force infiltration of SOSG with syringe into the leaf tissues through a hole exerted by a needle leads to uneven distribution of the fluorescent probe limited to a leaf mesophyll enclosed by vascular bundles. Furthermore, ^1^O_2_ is largely formed at the site of hole due to the mechanical injury. The observation that force infiltration of SOSG with syringe is inappropriate is in agreement with previous finding that pinhole infiltration maintains SOSG penetration into the epidermal cells which is unsuitable for study of high light stress^23^. Pressure infiltration using the shut syringe of leaf pieces was found to be both efficient in the uniform distribution of SOSG in the whole sample and unaffected by ^1^O_2_ formation due to the mechanical injury.

### SOSG photosensitization

Both SOSG and SOSG-EP were shown to serve as photosensitizers at visible light range^25–27^. In this reaction, singlet excited SOSG formed by excitation of SOSG by visible light passes to the triplet excited SOSG by a change in the orientation of the spin of excited electron via intersystem crossing. Triplet-triplet energy transfer from a triplet excited SOSG to molecular oxygen results in the formation of ^1^O_2_ and ground state SOSG. Due to these considerations, the relevance of pioneering works published need to be reconsidered cautiously as it might be possible that part of the ^1^O_2_ was produced by SOSG and SOSG-EP itself.

#### SOSG photosensitization in high-light treatment

Our results demonstrated that SOSG kept in dark or exposed to red light emits only negligible SOSG-EP fluorescence compared to white light (Fig. 8). Similarly, when Synechocystis cells (Fig. 9), Chlamydomonas cells (Fig. 10) and Arabidopsis leaves (Fig. 11) were exposed to white light, pronounced enhancement in SOSG-EP fluorescence was observed as compared to red light. These results demonstrate that a significant increase in SOSG-EP fluorescence upon illumination of SOSG with white light is caused by SOSG and SOSG-EP photosensitization. It is proposed that red light led to the formation of ^1^O_2_ by triplet-triplet energy transfer from excited chlorophyll to molecular oxygen, while white light caused the overall ^1^O_2_ formation due to the triplet-triplet energy transfer from excited chlorophyll and excited SOSG/SOSG-EP to molecular oxygen.

#### SOSG photosensitization during data collection

Imaging of SOSG and SOSG-EP fluorescence signal is mostly provided by confocal systems employing a 488 nm laser for excitation. However, the complex part of the microscopic study is finding the site of interest within a biological sample. Several authors^23,25^ registered an increase of SOSG fluorescence upon exposure to visible radiation. Epifluorescence microscopy of SOSG also utilizes visible light filters but the artefactual SOSG-EP fluorescence can be diminished by a standardized imaging design in which observations under a proper filter, in our case U-MNB-2 (excitation 470–490 nm, emission 520-IF, dichroic at 500 nm), are limited to a minimum time necessary for the only fast sample navigation and focusing.

### SOSG imaging and post-processing techniques

Synechocystis is characterized by a prokaryotic cell type (i.e. without internal compartmentalization) which size changes over the cell cycle; cells from the logarithmic and stationary phase of growth curve usually reach 1.5–2.5 µm in diameter^50^. Confocal laser scanning microscopy enables detection of the signal from a single focal plane and therefore imaging of more cyanobacterial cells in a single figure might be confusing as it usually includes also cells out of focus. In Chlamydomonas, the presence of flagella exerting algal movement hampers the relatively long confocal imaging. Unfortunately, the requirement of intactness of the cell for *in vivo* studies excludes the use of heat or chemicals used for flagella release. Thus, for unicellular organisms (cyanobacteria, algae) small volumes (ca 5–10 µl under cover slide) of the treated cell suspension can be advised for their immobilization throughout the microscopic observation. To study the 3-D distribution of SOSG-EP fluorescence within cells or tissues, Z-stacks of thin optical sections are employed in confocal microscopy. However, the time vs. resolution during scanning vs. number of optical sections must be compromised to avoid photobleaching. The mode how studied biological matrices affect the outcome of SOSG staining was compared in the three photosynthetic models and SOSG proved to be rather stable, i.e. suitable for multi-layer scans. To verify signal location, particularly in small objects like prokaryotic cells, it is advisable to decrease the pinhole size to 0.9–0.5 airy units, according to signal intensity. Based on the results when SOSG-EP fluorescence was detected in almost all optical sections of the various photosynthetic samples, ranging from unicellular prokaryote Synechocystis to complex tissue structure in Arabidopsis leaves, it can be pointed out that SOSG does not suffer from a significant photobleaching compared to other fluorescein probes.

Once collected the whole set of microphotographs (taken under the same standardized conditions) can be post-processed in suitable software to optimize signal-to-noise ratio and thus unable more clear presentation of the signal on screen or in print, particularly for figures with merged channels. This can help especially in 12-bit and 16-bit images, characterized by 4,096 and 65,536 shades, respectively. A universal approach cannot be advised due to the variability of plant materials but the crucial point is to apply the identical algorithm to all variants compared in the experiment to avoid false positive signals. Especially in multi-celled tissues, like Arabidopsis leaf sections, the signal location in chloroplasts is better visible after filtering the background and highlighting the SOSG signal in merged figures (Fig. 5B).

## Conclusions

The data presented in this study are expected to contribute to better understanding of the limitation in the use of SOSG for ^1^O_2_ detection in the photosynthetic organism. Although SOSG penetration into the cell and photosensitization of SOSG by visible light entails the main limitation of SOSG applicability in studies focused on high light stress in the photosynthetic organism, awareness of all the previously mentioned criteria (suitable infiltration procedure and red light) may lead to credible results. When measured under these conditions, SOSG can attend as highly sensitive and specific probe to identify the site of ^1^O_2_ formation in unicellular photosynthetic organisms and higher plants. However, quantitative analysis of ^1^O_2_ formation performed exclusively based on SOSG-EP fluorescence images is misleading. Only combination of several methods, e.g. SOSG-EP fluorescence imaging *in vivo* and spin probe EPR spectroscopy *in vitro*, provides comprehensive spatiotemporal and quantitative characterization of ^1^O_2_ formation in photosynthetic organisms. The different level of ^1^O_2_ production in different organism can also be attributed to variable cell density, different chlorophyll content, antioxidant level, within the cell/tissues etc. and thus based on the parameters to be evaluated, proper control experiments are required.

## Electronic supplementary material

Supplementary Dataset 1–5
