# Supplementary material for: Singlet oxygen imaging using fluorescent probe Singlet Oxygen Sensor Green in photosynthetic organisms
Source: Sci Rep. 2018 Sep 12;8:13685. doi: 10.1038/s41598-018-31638-5 (PMC6135792; doi:10.1038/s41598-018-31638-5)
Supplement: Supplementary file 1 — Supplementary Dataset 1–5 [file 41598_2018_31638_MOESM1_ESM.pdf]

Singlet oxygen imaging using fluorescent probe Singlet Oxygen Sensor Green in photosynthetic organisms

Ankush Prasad[1]*, Michaela Sedlářová[2]*, Pavel Pospíšil[1#]

*The author's contributed equally to the manuscript

[1]Department of Biophysics, Centre of the Region Haná for Biotechnological and Agricultural Research, Faculty of Science, Palacký University, Šlechtitelů 27, 783 71 Olomouc, Czech Republic
[2]Department of Botany, Faculty of Science, Palacký University, Šlechtitelů 27, 783 71, Olomouc, Czech Republic

[#]**Corresponding author**
E.mail: pavel.pospisil@upol.cz

Supplementary Fig. S1

Synechocystis PC6803

DIC | SOSG-EP | DIC+SOSG-EP | SOSG-EP intensity

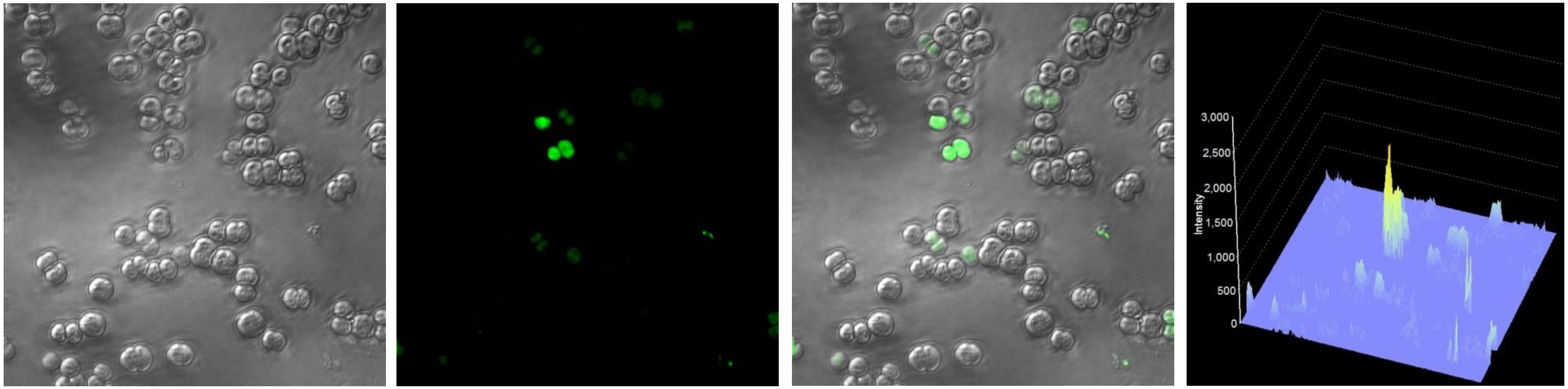

Biological replicate 2

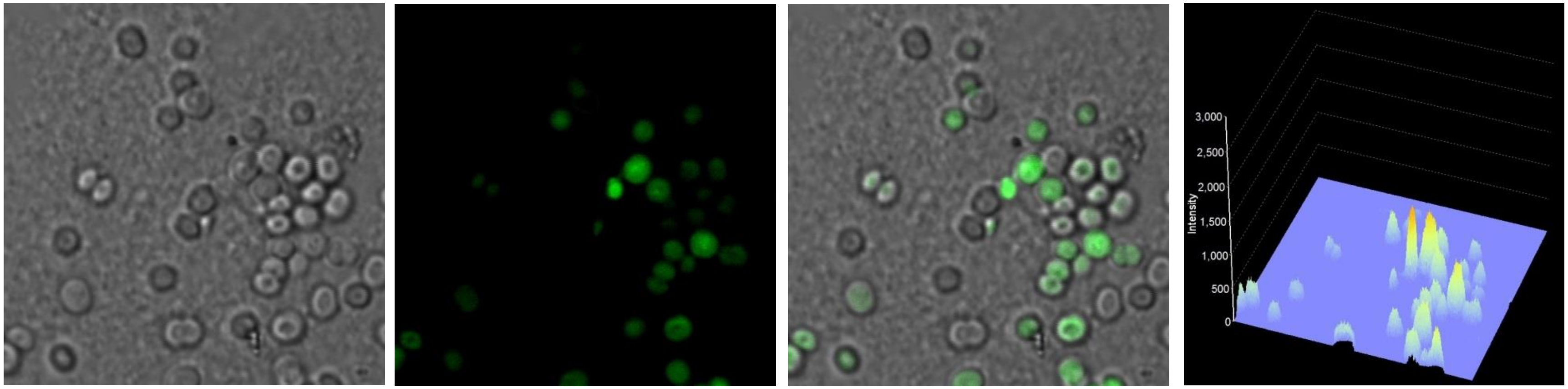

Biological replicate 3

Figure S1: Biological replicates on effect of red light on singlet oxygen imaging in Synechocystis cells. Synechocystis cells were treated in 50 mM SOSG for 30 min at 37°C. For each treatment following images are presented (from left to right): Nomarski DIC, SOSG-EP fluorescence (λem = 505–525 nm), combined channel and integral distribution of the signal intensity within the sample (Z-axis represents the levels of brightness for each pixel, ranging between 0 and 3000).

Supplementary Fig. S2

Chlamydomonas reinhardtii

DIC | **SOSG-EP** | **DIC+SOSG-EP** | **SOSG-EP intensity**

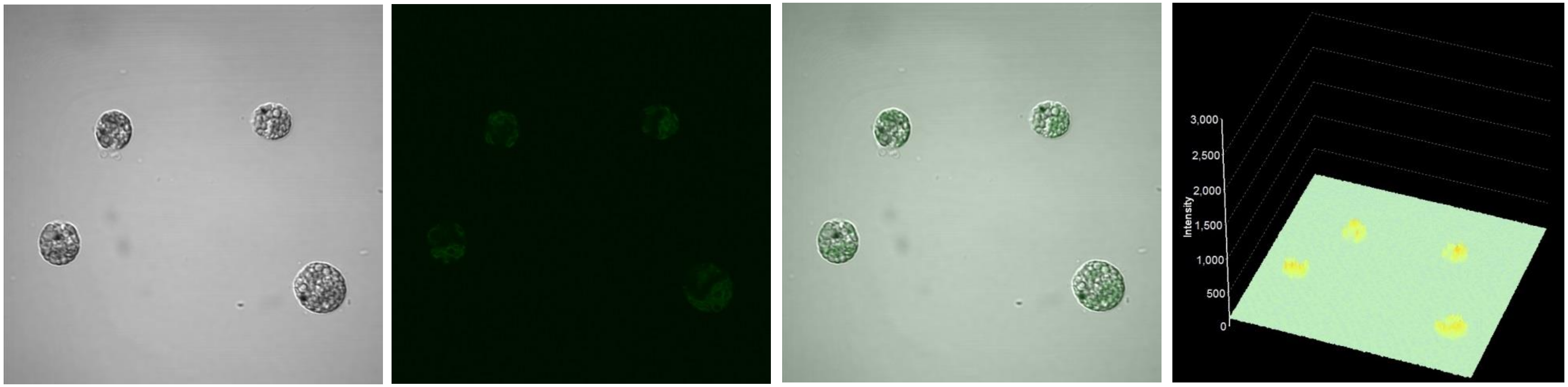

Biological replicate 2

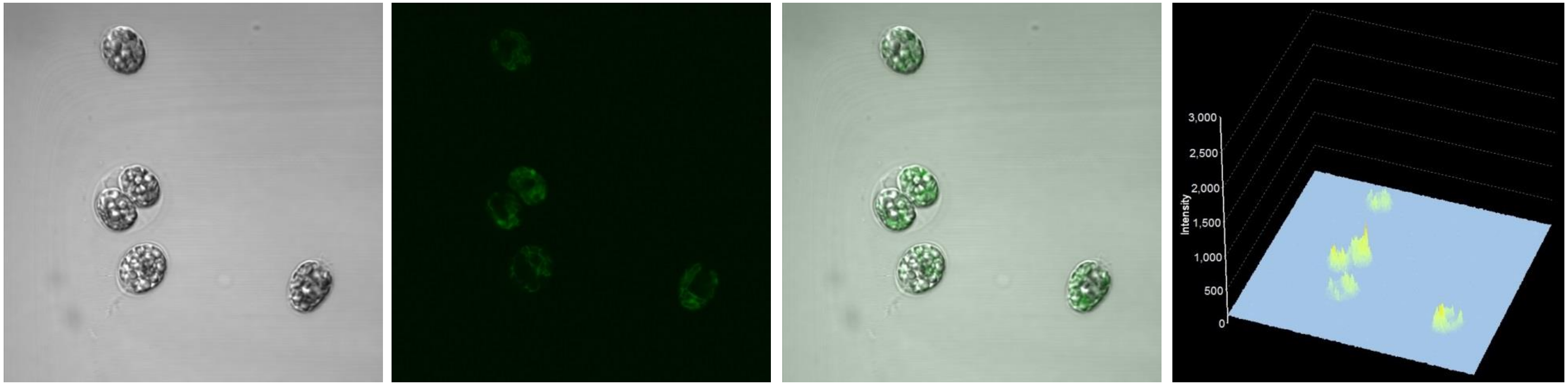

Biological replicate 3

Figure S2: Biological replicates on effect of red light on singlet oxygen imaging in Chlamydomonas cells. Chlamydomonas cells were treated in 50 mM SOSG for 30 min. For each treatment following images are presented (from left to right): Nomarski DIC, SOSG-EP fluorescence (λem = 505–525 nm), combined channel and integral distribution of the signal intensity within the sample (Z-axis represents the levels of brightness for each pixel, ranging between 0 and 3000).

Supplementary Fig. S3

Arabidopsis thaliana

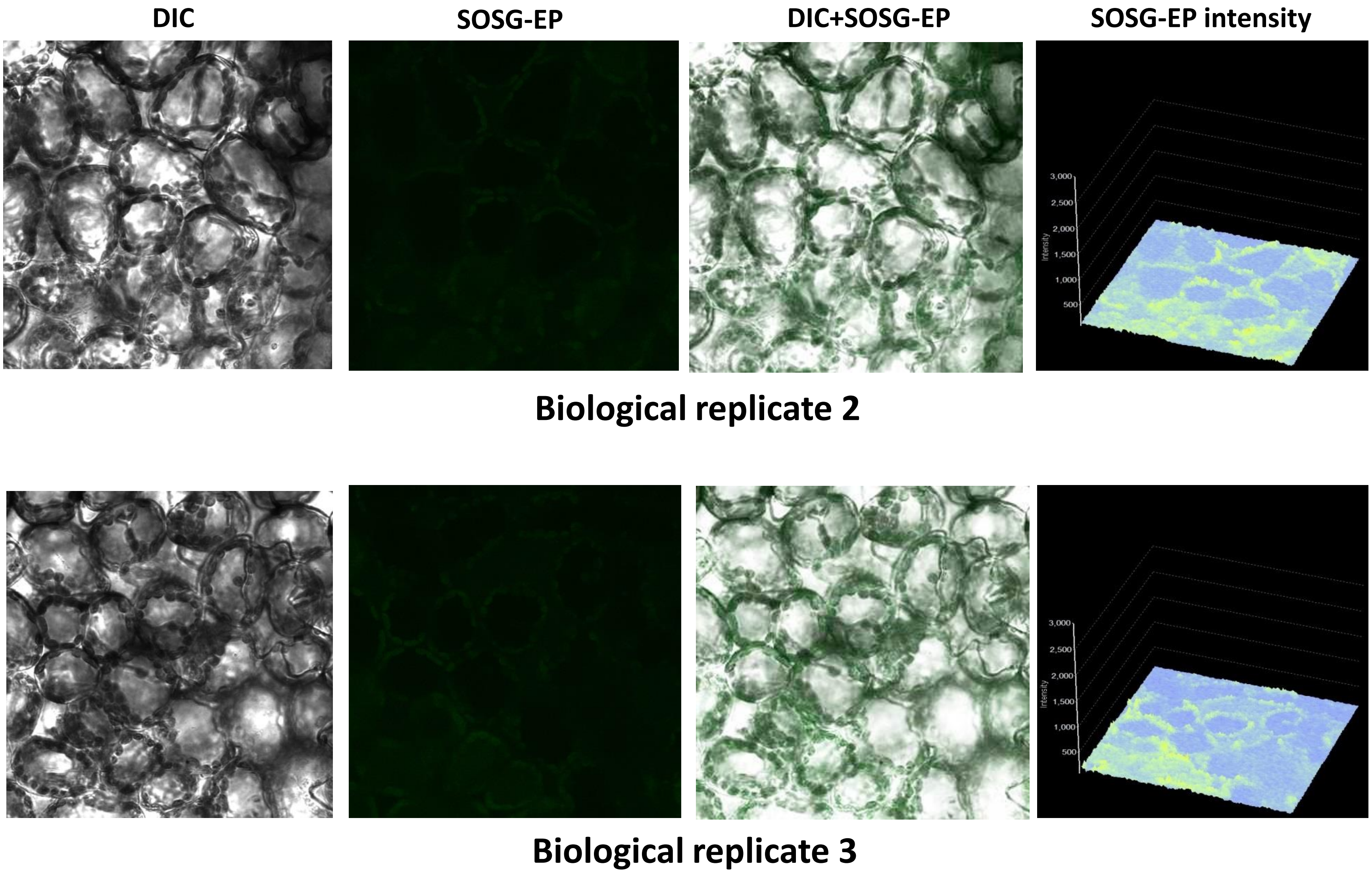

Figure S3: Biological replicates on effect of red light on singlet oxygen imaging in Arabidopsis leaves. Arabidopsis leaves were treated in 50 mM SOSG for 30 min. For each treatment following images are presented (from left to right): Nomarski DIC, SOSG-EP fluorescence (λem = 505–525 nm), combined channel and integral distribution of the signal intensity within the sample (Z-axis represents the levels of brightness for each pixel, ranging between 0 and 3000).

Supplementary Fig. S4

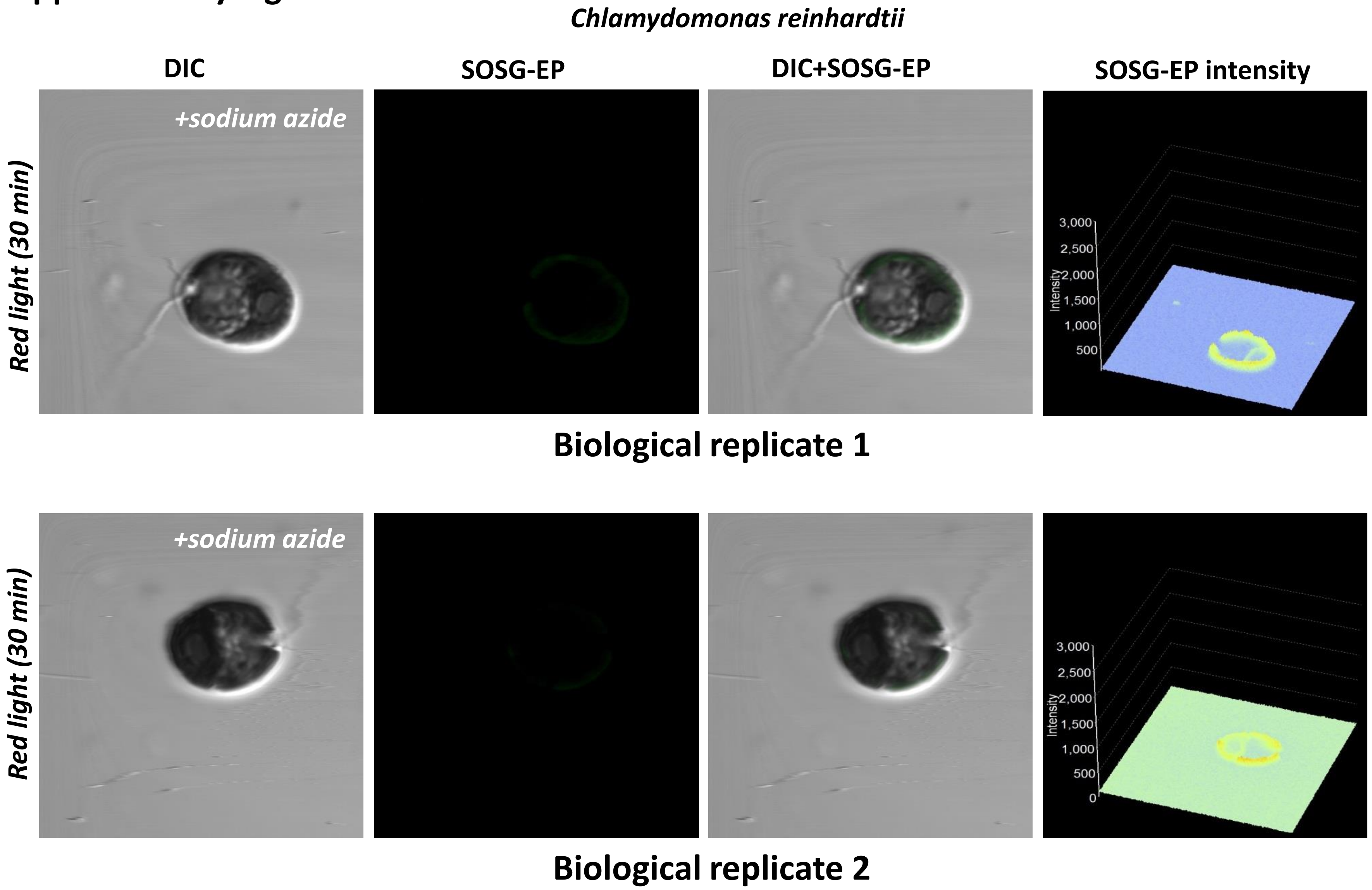

Figure S4: Singlet oxygen imaging in Chlamydomonas cells and effect of scavengers. Chlamydomonas cells were treated in 50 mM SOSG for 30 min in the presence of 0.1% sodium azide (NaH). For each treatment following images are presented (from left to right): Nomarski DIC, SOSG-EP fluorescence (λem = 505–525 nm), combined channel and integral distribution of the signal intensity within the sample (Z-axis represents the levels of brightness for each pixel, ranging between 0 and 3000).

Supplementary Fig. S5

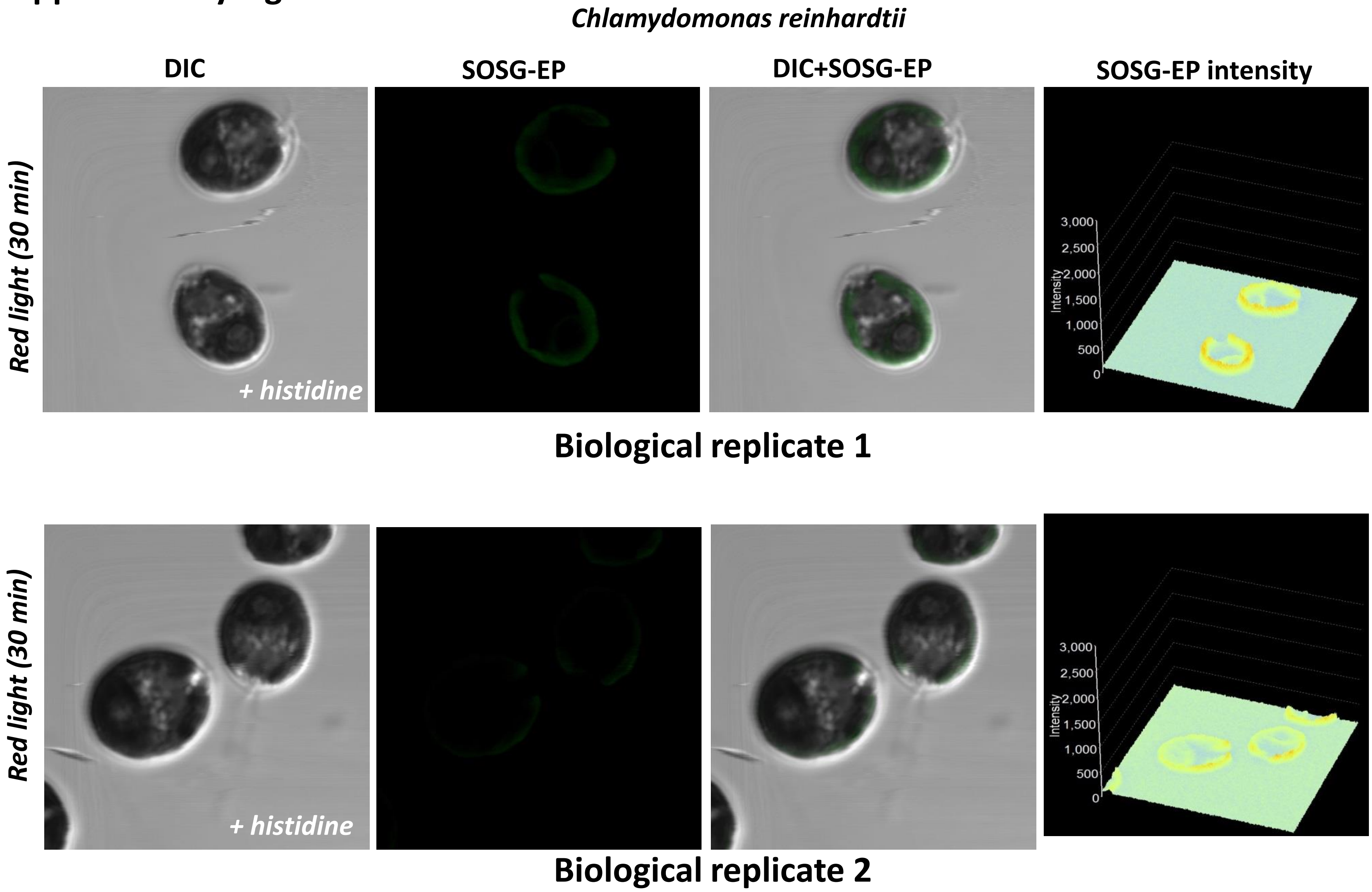

Figure S5: Singlet oxygen imaging in Chlamydomonas cells and effect of scavengers. Chlamydomonas cells were treated in 50 mM SOSG for 30 min in the presence of 500µM Histidine. For each treatment following images are presented (from left to right): Nomarski DIC, SOSG-EP fluorescence (λem = 505–525 nm), combined channel and integral distribution of the signal intensity within the sample (Z-axis represents the levels of brightness for each pixel, ranging between 0 and 3000).
